# Modelling of pancreatic cancer biology: transcriptomic signature for 3D PDX-derived organoids and primary cell line organoid development

**DOI:** 10.1038/s41598-020-59368-7

**Published:** 2020-02-17

**Authors:** Shannon R. Nelson, Chenxi Zhang, Sandra Roche, Fiona O’Neill, Niall Swan, Yonglun Luo, AnneMarie Larkin, John Crown, Naomi Walsh

**Affiliations:** 1 National Institute for Cellular Biotechnology, School of Biotechnology, Dublin City University, Dublin 9, Ireland; 2 BGI Education Centre, University of Chinese Academy of Sciences, Shenzhen, 518083 China; 3 Lars Bolund Institute of Regenerative Medicine, BGI-Qingdao, BGI-Shenzhen, Qingdao, 266000 China; 4 St Vincent’s University Hospital, Elm Park, Dublin 4, Ireland; 5 Institute of Technology, Sligo, Ash Lane, Sligo, Ireland; 6 Department of Biomedicine, Aarhus University, 8000 Aarhus, Denmark

**Keywords:** Cancer models, Cancer models

## Abstract

With a five-year survival rate of 9%, pancreatic ductal adenocarcinoma (PDAC) is the deadliest of all cancers. The rapid mortality makes PDAC difficult to research, and inspires a resolve to create reliable, tractable cellular models for preclinical cancer research. Organoids are increasingly used to model PDAC as they maintain the differentiation status, molecular and genomic signatures of the original tumour. In this paper, we present novel methodologies and experimental approaches to develop PDAC organoids from PDX tumours, and the simultaneous development of matched primary cell lines. Moreover, we also present a method of recapitulating primary cell line cultures to organoids (CLOs). We highlight the usefulness of CLOs as PDAC organoid models, as they maintain similar transcriptomic signatures as their matched patient-derived organoids and patient derived xenografts (PDX)s. These models provide a manageable, expandable *in vitro* resource for downstream applications such as high throughput screening, functional genomics, and tumour microenvironment studies.

## Introduction

With a rapid progression and fatal outcome, pancreatic cancer is one of the deadliest of all cancers. Long-term survivors are limited to those with resected early stage tumours; however, overall survival rate is a dismal 9%, with a median survival time of 7–11 months^1,2^. As pancreatic cancer is notoriously asymptomatic at an early stage, 80% of patients are diagnosed after the cancer has metastasised, making them ineligible for resection, which is the only curative treatment. The number of cases of pancreatic cancer has been steadily rising since 2004^3^. It is currently the fourth most common cause of cancer death in the US, and by 2030 it is estimated that it will surpass breast and colorectal cancer to become the second most common cause of death by cancer^4^. The majority of pancreatic cancers are in the exocrine pancreas (95%) known as pancreatic ductal adenocarcinoma (PDAC), and 5% are in the endocrine pancreas^5^. The leading epidemiological factors include smoking, obesity, type II diabetes mellitus and acute pancreatitis, which account for approximately 25% of PDAC cases^6–9^.

A limitation in the understanding of the disease progression and development of effective treatments in PDAC may be due the lack of *in vitro* patient tumour representative models. Established 2D cell lines are the most widely used model for the development and testing of chemotherapeutics for over 50 years^10^. The ability of cell lines to grow indefinitely makes them a low-cost, repeatable model, easy to manipulate and are an important base for discovery and proof-of-concept studies. Their importance in cancer research is indisputable, however, their use as a robust clinical model is questionable^11^. During passaging, cell lines undergo genetic modifications, such as copy number variation and point mutations^12^. Cell lines also have a high level of homogeneity, which does not represent the heterogenetic nature of PDAC tumours, and not all cancer subtypes are well represented as they are usually developed from aggressive and metastatic tumours, therefore tumour progression cannot be studied^13^. Deer *et al*.^14^ highlighted the genetic diversity and differences in adhesion, migration and tumorgenicity between the different PDAC cell lines in a detailed review of phenotypes and genotypes of PDAC cell lines. Primary tumour cultures derived from patient tumour or biopsy and grown *in vitro* in 2D tend to be heterogeneous, and are a better representation of the tumour of origin tissue as the cells are at an early passage number^15^. These models allow for the development of personalised cancer therapy as demonstrated through functional screening of chemotherapeutic drugs, modelling individual tumour response^16^. However, although an important tool in cancer research, there are limitations associated with primary cell culture, including the difficulty of obtaining tumour samples. Primary cell cultures are often difficult to establish as the initial sample may lack tumour cells; the outgrowth of stromal cells such as fibroblasts may overrun the culture, and the cells may only grow for a finite number of passages^15,16^.

Three-dimensional (3D) *in vitro* models can address the limitations of growing cancer in 2D. Unlike 2D cultures, 3D cell cultures are not grown attached to plastic, so they adopt a more physiologically relevant morphology and signalling pathways similar to *in vivo* conditions^17^. Like solid tumours, 3D cell cultures are exposed to complex physiological and heterogeneous environments, resulting in varied exposure to oxygen, nutrients and stresses. This allows for the study of cell-to-cell interaction, drug penetration, response and resistance^18,19^. The 3D culture model contains proliferating, quiescent, hypoxic and necrotic cells, whereas in 2D cell models, all the cells are in the same growth phase^20^. Organoids are 3D cultures derived from organ specific stem cells, allowing for the self-organisation of cells to resemble structures from within an organ or tumour^21^. These 3D system models the physiology, shape, dynamics and cell make-up of the cancer and produces a relevant and highly adaptable model system^22^. Organoids can be derived from embryonic stem cells, induced pluripotent stem cells, and both tumour and normal organ restricted adult stem cells (aSCs)^21^. Organoids can be maintained in a 3D matrix which supports cell growth, such as Matrigel or hydrogels which are laminin rich, mimicking the pancreatic microenvironment *in vivo*^23^. Organoid culture requires special media containing a cocktail of growth factors that mimic the microenvironments of organ stem cell niches^24^. Unlike established and primary cell lines, organoids display cell heterogeneity after several passages and organoids which have been developed from the pancreas can be expanded, managed and cryopreserved and PDAC organoids can proliferate indefinitely^22^. Boj *et al*.^25^ developed a method for the establishment of both normal and neoplastic organoids from human and murine pancreatic tissues. Orthotopic transplant of both the PDAC and normal organoids resulted in full tumour and ductal development respectively. These methods are useful for the further development of PDAC research, including the study of the tumour microenvironment, personalised medicine, furthering the understanding of the genetics and for testing novel therapeutics in PDAC.

Patient derived xenografts (PDX) are widely used animal models to study cancer. The patient tumour is grown subcutaneously or orthotopically in an immunodeficient mouse, and sub-cultured into new mice when the tumour reaches a sufficient size. PDX models allow for tumours to retain their cell-to-cell interactions, and orthotopic implantation allows for the tumour to grow in the same micro-environment as the original tumour^26^.

There are several advantages to using PDX tumours for the study of PDAC. PDX tumours are established from small amount of patient tumour; the tumours retain the original tumour heterogeneity, which can be lost in established cell lines. The PDX tumours also preserve the original tumours’ genetics and histological characteristics during passaging. These models are used as an unlimited resource for *in vivo* and *ex vivo* drug testing, which is invaluable in PDAC research. However, disadvantages to the use of PDX models include: expensive to develop, time consuming, dependent on the use of animals, require ethics approval and are subject to strict regulations^27^. PDX models also have a slow take rate, and can take months to develop tumours. Moreover, as the tumour is sub-cultured, the tumour-associated stroma is replaced by murine stromal cells, such as blood vessels and fibroblasts^28^. Finally, SCID-mice which are used for PDX biobanks lack immune systems, and this limits the testing of drugs such as immune modulators, which are increasingly being used in cancer treatments.

PDX models and 3D cancer organoid cultures are important preclinical *in vitro* live material models. However, limitations for both include considerable amount of time required and cost to expand cultures *in vitro*, which makes it difficult to scale-up and perform downstream high‐throughput analysis. Here, we report methodologies for the establishment of PDX-tumour organoids, isogenetically matched 2D primary cell line and recapitulated primary cell line into organoids (CLOs). We show that organoids and CLOs maintain the same molecular and transcriptomic signatures compared to the 2D primary cell line, indicating the robustness and usefulness of CLOs in preclinical studies of pancreatic cancer.

## Results

### Development of PDX organoids, isogenetically matched primary cell lines and 3D cell line organoids (CLOs) as novel models to study PDAC

**Figure 1:**
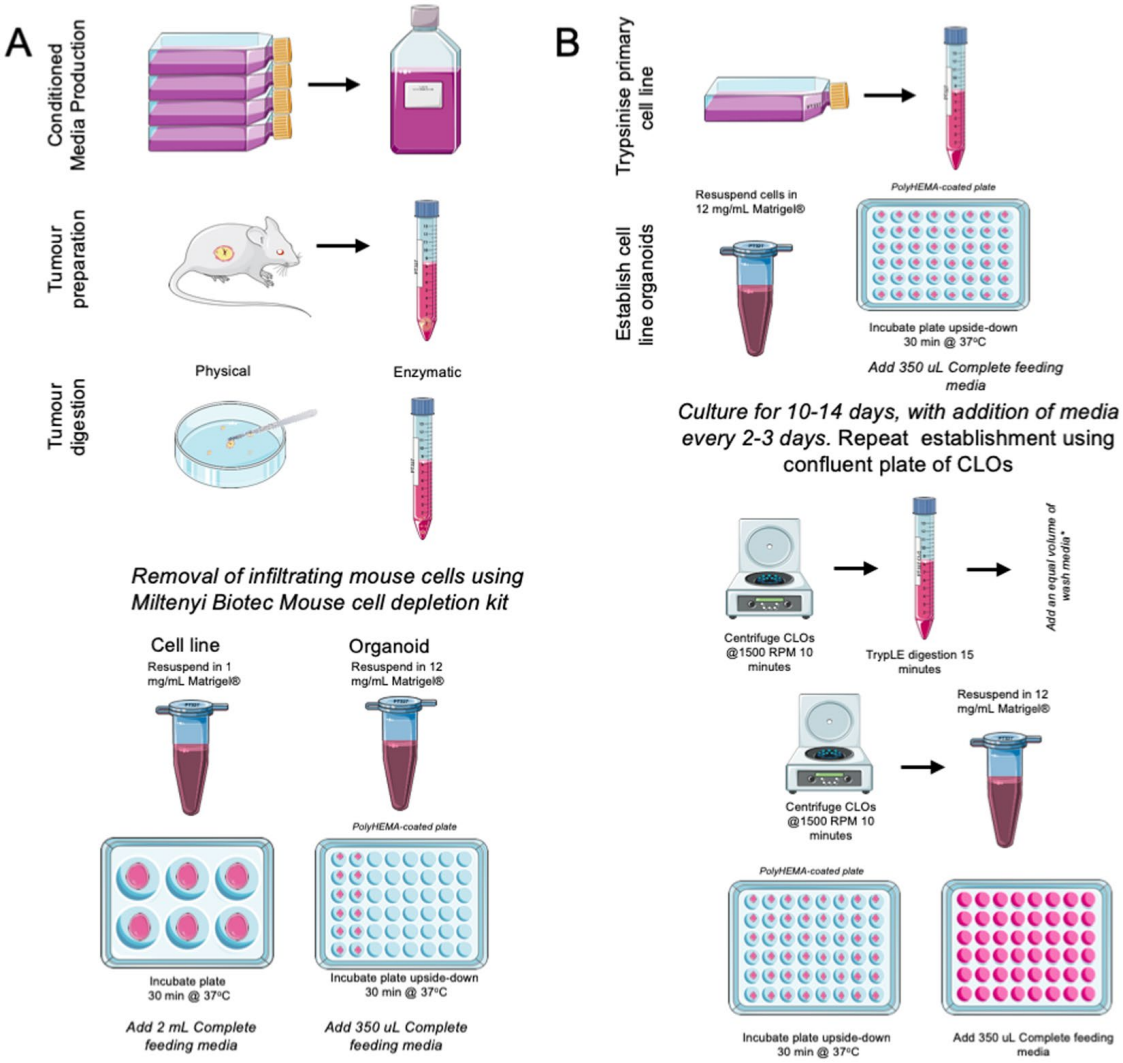
Schematic diagram protocol for generating PDAC organoids, isogenetically matched 2D primary cell lines and cell line organoids (CLOs) from a primary cell line. (**A**) The steps involved in the simultaneous production of organoids and isogenetically matched 2D primary cell lines from a single PDX tumour sample, (**B**) Steps involved in the establishment of CLOs from a 2D primary cell line. This figure was created using Servier Medical Art templates, which have been modified. These images are licensed under a Creative Commons Attribution 3.0 Unported License; https://smart.servier.com.

**Figure 2:**
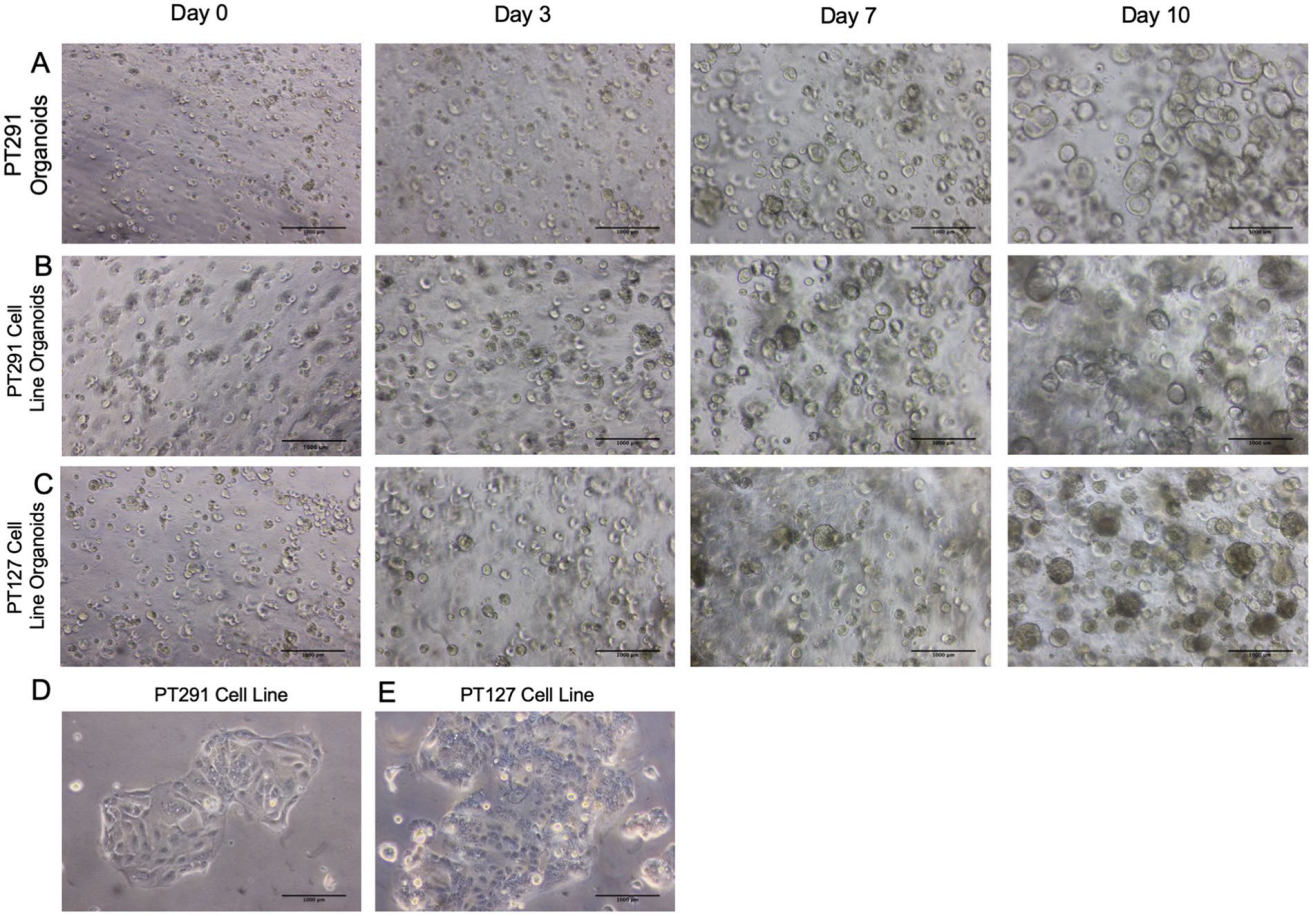
Brightfield images at Day 0, Day 3, Day 7, Day 10 of (**A**) PT291 organoids (**B**) PT291 CLO (**C**) PT127 CLO (**D**) PT291 2D primary cell line and (**E**) PT127 2D primary cell line. Scale bars 1000 µm.

Recent progress with preclinical models to study PDAC has advanced our research into this deadly disease. We have built on the scientific advancements in PDX^28–30^ and cancer organoid models^21,25,31,32^ to bridge the *in vivo*/*in vitro* gap in PDAC research. Therefore, we developed a protocol to established organoids from PDX tumour with modified approaches from Boj *et al*. (Fig. 1A)^25^. Primary cell lines were simultaneously generated by maintaining organoid outgrowth cells in lower concentration extracellular matrix (ECM) gel derived from Engelbreth-Holm-Swarm murine sarcoma on adherent wells, and allowing the cells to attach and proliferate. This method allows for the rapid development of primary cell lines, which can be scaled up and manipulated. The 2D primary cells are cultured for two passages then recapitulated as 3D cultures (Fig. 1B) and embedded in high concentration ECM, which provides a scaffold for PDAC organoid growth. This novel method leads to the rapid development of cell line organoids (CLOs), which are morphologically similar to the matched organoids within only 3–7 days (Fig. 2A–C).

### Confirmation of primary cell lines and organoid culture derived from human PDAC PDX tumour cells

**Figure 3:**
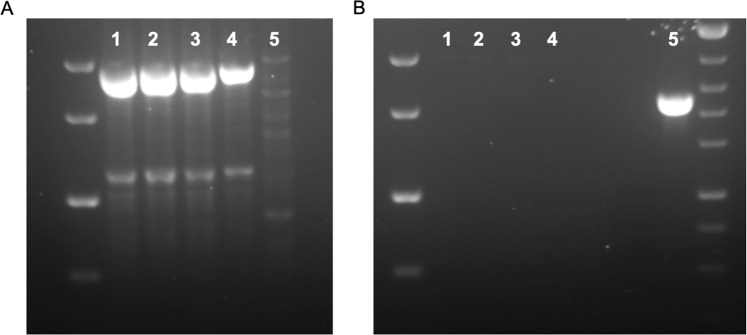
Electrophoresis gels of samples (1) PT291 primary cell line (2) PT127 primary cell line (3) PT291 organoids (4) BxPC3 (negative control) (5) Murine cell line C2C12 (positive control) for (**A**) Products of first PCR and (**B**) Nested PCR using mouse-specific primers.

Limitations of the use of PDX tumours in cancer research is infiltrating mouse stroma cells into the PDX tumour. A mouse cell depletion kit was used for the removal of the infiltrating mouse cells. A two step, nested PCR method was used to confirm the absence of mouse cells in the primary cell lines and organoids^33^. Universal primers amplified a region common in both the human and mouse mitochondrial genomes (Fig. 3A). Mouse specific primers were used to identify if mouse-contaminating cells were present in the samples. Figure 3B confirms that no contaminating mouse mitochondrial DNA is present in the PDX derived organoids, 2D primary cell lines and CLOs.

### Confocal microscopy reveals CLO models representative of derived organoid

**Figure 4:**
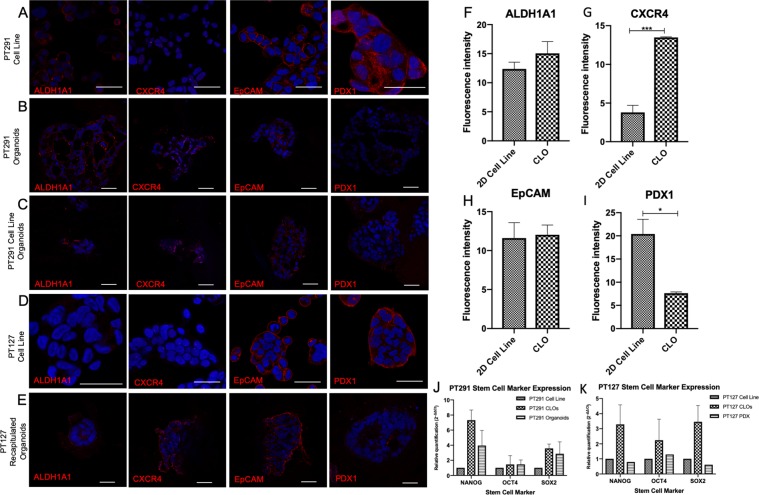
Confocal images of immunofluorescence staining images of ALDH1A1, CXCR4, EpCAM, and PDX1 (red) scale bars 40 μm in (**A**) PT291 2D primary cell line (**B**) PT291 organoids (**C**) PT291 CLO (**D**) PT127 2D primary cell line and (**E**) PT127 CLO. DAPI counterstain (blue). Images are representative of three independent experiments. (**F–I**) Analysis comparing the fluorescence intensity between PT291 CLOs and 2D cell lines in (**F**) ALDH1A1 (**G**) CXCR5 (**H**) EpCAM and (**I**) PDX1. (**J,K**) Relative quantification of stem cell markers *NANOG*, *OCT4* and *SOX2* assessed using qRT-PCR in (**J**) PT291 PDX derived cell line, organoids and CLOs, and (**K**) PT127 PDX derived cell line, CLOs and PDX tumour sample. Expression is shown relative to the expression in matched cell line. 18 S rRNA used as endogenous control n = 3.

Confocal immunofluorescence was performed in order to determine whether the expression profile of key PDAC tumour initiating markers was maintained in organoids and CLOs. Organoid cultures and derived CLOs were enriched for ALDH1A1 and CXCR4 populations and displayed decreased expression of PDX1 compared to 2D primary cell lines; whereas a similar expression profile for EpCAM was observed in all samples (Fig. 4A–E). To further test for the presence of stem cell markers responsible for self-renewal and propagation in the organoids and CLOs, quantitate PCR (qPCR) was performed. Expression of specific markers *OCT4*, *NANOG* and *SOX2* was increased in PT291 organoids and CLOs compared to the 2D primary cell lines (Fig. 4J), while PT127 PDX tumour had a similar expression profile to the PT127 CLOs compared to its 2D primary cell line (Fig. 4K).

### RNA-seq transcriptomic analysis identifies novel signature defining recapitulated CLO

**Figure 5:**
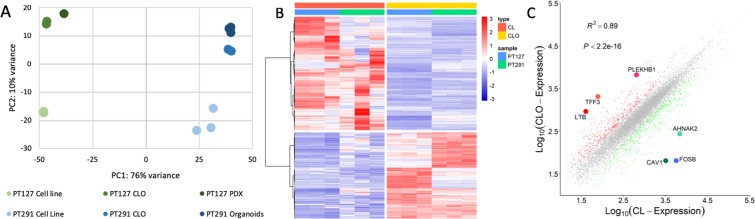
(**A**) Principal component analysis was performed to compare the expression profiles of PT127 Cell line, PT127 CLO, PT127 PDX, PT291 cell line, PT291 CLO and PT291 organoids (**B**) Clustering of all differentially expressed genes (fold change >1, p-adj = 0.001) in CLOs and cell lines (PT127 and PT291) (**C**) Scatterplot – grouped PT291 and PT127 CLO vs CL, genes with the biggest log fold-change highlighted. FOSB (log fold change −6.496, p-adj 6.65E-50), CAV1 (log fold change −5.7652 p-adj 4.89E-75), AHNAK2 (log fold change −4.7934931, p-adj 9.25E-20), TFF3 (log fold change 4.8678, p-adj 5.86E-32), PLEKHB1 (log fold change 3.803, p-adj 2.59E-13), LTB (log fold change 3.633, p-adj 2.32E-11).

RNA-seq was performed to identify transcriptome signatures of genes dysregulated among PDX organoids/PDX tumour and corresponding CLOs compared to 2D primary cell lines. Principal component analysis (PCA) revealed PT291 organoids and CLOs clustered together; while PT127 PDX tumour and CLOs clustered together, separate from their corresponding isogenetically matched 2D primary cell line (Fig. 5A). Hierarchical clustering was performed of all differentially expressed genes in the grouped CLOs and 2D primary cell lines (PT291 and PT127) (Fig. 5B).

In Fig. 5C, a scatterplot reveals the most differentially expressed genes between grouped PT291 and PT127 CLOs compared to grouped PT291 and PT127 cell line. We identified *FOSB* (log fold change −6.496, p-adj 6.65E-50), *CAV1* (log fold change −5.7652 p-adj 4.89E-75) and *AHNAK2* (log fold change −4.7934931, p-adj 9.25E-20) as the three top down regulated genes in the grouped CLOs compared to the grouped 2D primary cell lines. *TFF3* (log fold change 4.8678, p-adj 5.86E-32), *PLEKHB1* (log fold change 3.803, p-adj 2.59E-13) and *LTB* (log fold change 3.633, p-adj 2.32E-11) were the top up regulated genes.

**Figure 6:**
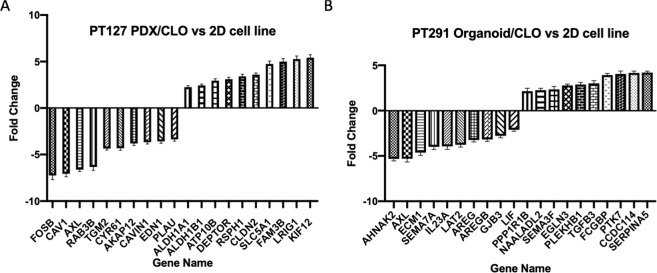
(**A**) The 10 most significant (p-adj ≤ 7.15E-42) up and down regulated genes in PT127 CLO/PDX compared to the matched 2D cell line (**B**) The 10 most significant genes in PT291 CLO/organoid vs 2D cell line.

To further identify a specific transcriptomic signature common among the PDX tumour/organoid and CLOs we assessed the transcriptomic signature of the PT127 PDX-derived tumour and CLO compared to the 2D primary cell line, and the PT291 organoid and CLO versus the 2D primary cell line. We observed 144 genes up regulated and 130 genes down regulated (log2 fold change ≥2, p-adj 0.001) in both PT127 PDX tumour/CLO compared to the isogenetically matched cell line in 2D (Supplementary Tables 6 and 7). In PT291, 62 genes were up-regulated and 105 genes down regulated in the organoids and CLOs compared to the isogenetically matched 2D cell line (Supplementary Tables 8 and 9). The 10-most significantly (p-adj ≤ 7.15E-42) dysregulated genes in PT127 PDX and CLO compared to the 2D cell line are shown in Fig. 6A; *KIF12* had the highest log2 fold change (log2 fold change 5.422, p-adj = 1.62E-62) and *FOSB* (log2 fold change −7.241, p-adj 1.82E-55) had the lowest fold change. The 10 most significant (p-adj ≤ 5.79E-14) up and down-regulated genes in PT291 organoids and CLOs compared to the isogenetically matched 2D cell line are outlined in Fig. 6B. *SERPINA5* (log2 fold change 4.212, p-adj = 2.17E-14) had the highest log2 fold change, and *AHNAK2* (log2 fold change −5.316, p-adj = 1.28E-110) displayed the lowest fold change.

## Experimental Design

The method for the establishment of PDAC PDX organoids and isogenetically matched cell lines was modified from Boj *et al*.^25^. Cultures were prepared as outlined below: a mouse cell depletion kit for the removal of the infiltrating mouse cells in the PDX samples, with a PCR based method to confirm the removal of the mouse cells^33^. Wnt3a, Rspondin3 and Noggin conditioned media was made using cell line L-WRN (ATCC, CRL-3276), and was assessed for the levels for protein using the TOPFlash dual luciferase assay^34^.

### Patient demographics and PDX information

PDAC primary tumour tissue specimens were obtained from Saint Vincent’s University Hospital Ethics and Medical Research Committee, Dublin, Ireland between 2013 and 2017. All patients underwent pancreatic adenocarcinoma resection. All donors agreed by written informed consent to donate tissue and for study participation. The specimen donor’s personal information was confidential and protected except the date of birth. All samples and methods used in this study were approved by Saint Vincent’s University Hospital Ethics and Medical Research Committee and conducted in accordance with the relevant guidelines and regulations in compliance with the Saint Vincent’s University Hospital Ethics and Medical Research Committee. PDAC PDX tissues were generated by subcutaneous seeding in severe combined immunodeficiency disease (SCID) mice. Primary patient samples were confirmed as PDAC by a pathologist (N.S.) in Saint Vincent’s University Hospital, and PDX samples were also confirmed by pathology examination to maintain the human tumour content and morphology of the original tumour.

All animal work has received ethical approval from the DCU Research Ethics Committee (DCUREC/2012/202) and was licensed by Department of Health (B100–4501) all methods were performed in accordance with the relevant guidelines and regulations in compliance with the DCU Research Ethics Committee and the Department of Health.

### PDX tumour development

After initial macroscopic pathological confirmation, material remaining after diagnostic sampling was cold transferred in RPMI media containing 1% Pen Step, 1% fungazone to DCU.

The tumour was cut into implant sized pieces (<2 mm) and rinsed with fresh serum-free RPMI media following transport. Severe combined immunodeficiency (SCID), CB17/lcr-Prkdc^scid^/lcrCrl mice (Charles River, UK) were implanted subcutaneously with fresh patient tumour material. Under anaesthesia (isoflourane, O_2_ carrier gas) a small incision was made in the skin of the left flank of the animal. The tumour piece was placed in the pocket under the skin and the wound sealed with a single staple. The animals were monitored post-surgery, and staple removal was within 10 days. Animals were monitored weekly for body weight and tumour development. Mice were monitored for tumour development for up to 1 year post implantation. Animal welfare monitoring criteria included tumour volume, tumour axis, body weight and condition, where maximum tumour volume is <2000 mm^3^, and maximum tumour axis is <20 mm. A decrease in body weight of >10% resulted in increased monitoring, with body weight decrease of 20% resulting in humane euthanasia. Tumour measurements were by calliper measurement and tumour volume was calculated as (HxDxW)/2, where H indicated height, D indicated depth and W indicated width. At experiment end, the animals were humanely euthanized and the tumour was harvested. PDX samples were confirmed by pathology examination to maintain the human tumour content and morphology of the original tumour.

### PDX tumour preparation

The media for organoid establishment and culture is represented in Supplementary Tables 1–4. Firstly, a 21 mL solution of 20:1 PBS to PB Buffer (Miltenyi Biotec, 130-091-376), and a 2% PenStrep in PBS solution was prepared. The PDAC PDX tumour sample was received on ice, in basal media. Using a Pasteur pipette, all media was removed and 10 mL of the PBS-PenStrep solution was added to the sample, and the tube was shaken for 20 seconds. The tumour sample was allowed to settle, and the PBS was aspirated. The process was repeated with 10 mL and 15 mL washes. In a petri-dish, the tumour was chopped finely using a scalpel and forceps. The sample was placed in a 50 mL Falcon tube, and 10 mL digestion buffer (Supplementary Table 3) was added. The tube was placed on a rocking table at room temperature for 15 minutes. The tumour sample was removed to a laminar flow hood. The digested tumour sample was put through a MACS SmartStrainers (70 µm) (Miltenyi Biotec, 130-098-462) using a cell scraper into a fresh 50 mL falcon tube. The cell strainer was rinsed with 20 mL wash media (Supplementary Table 2). The cells were centrifuged at 1500 RPM for 7 minutes at room temperature (RT).

### Mouse cell depletion kit

Following centrifugation, the media was aspirated, and the pellet was resuspended in 5 mL PB buffer, making a single cell suspension. Using Trypan blue (Sigma, T8154O), the cells were counted and a cell suspension of 2 × 10^6^ cells was centrifuged at 1500 RPM for 10 minutes at RT, and resuspended in 80 μL PB buffer, and 20 μL of the mouse cell depletion kit antibody cocktail (Miltenyi Biotec, 130-104-694). This suspension was incubated at 4 °C for 15 minutes. Following incubation, 400 μL of PB buffer was added to the cell suspension and mixed thoroughly. An LS column (Miltenyi Biotec, 130-042-401) was placed in the MidiMacs Separator (Miltenyi Biotec, 130-042-302) and rinsed with 3 mL PB buffer. The cell suspension was pipetted into the column, and the flow-through was collected in a 15 mL tube. The column was washed twice with 1 mL of PB buffer, and the flow-through was collected in the same tube. The cells were centrifuged at 1000 RPM for 5 minutes at 4 °C. All media was removed, using a P200 to avoid disrupting the pellet.

### Organoid establishment

The cells were resuspended in 50 μL ECM (Sigma, E1270, Lot 087M177V 8–12 mg/mL) per 1.5 × 10^5^ cells. 20 μL of organoid:12 mg/ml ECM solution was pipetted into poly-HEMA coated 48-well plate. The plate was placed in an incubator upside down at 37 °C for 20 minutes for ECM to polymerise. 350 μL of the Complete Human Feeding Media (CHFM) (Supplementary Table 4) with RhoK inhibitor was added to each well of the 48 well plate. Two days after establishing the tumour sample, cells were fed using CHFM with RhoK inhibitor, then subsequently fed using CHFM without RhoK inhibitor (Supplementary Table 4).

### Establishment of isogenetically matched 2D primary cell line from organoids

Protocol was followed as outlined above in “mouse cell depletion kit”. On day zero, 1.5 × 10^5^ cells were mixed in 100 μL of ECM diluted to 1 mg/mL and 50 μL was plated per well in a non-poly-HEMA coated 24 well plate. Cells were fed 500 μL of the CHFM with RhoK inhibitor (Supplementary Table 4). Cells were cultured as above for a further 10 days, or until the cells began to adhere to the bottom of the plate, feeding with CHFM. To passage cells, 1 mL TrypLE (Gibco, 12605010) was added to each well until the cells detached, and 1 mL of DMEM High-Glucose Glutamax (Gibco, 10566–016) with 10% FBS was added to stop trypsinisation. Cells were grown in a 6-well plate, then up scaled to T25 cm^3^ flask. Once in the T25 cm^3^ flask, the media was changed from CHFM to 50:50 GlutaMax DMEM and L-WRN Conditioned Media with 10% FBS and 1% PenStrep.

### Passaging organoids

A fresh poly-HEMA coated 48 well plate was preheated to 37 °C. The media from each well was removed to a 30 mL tube, and the well was washed with 300 μL ice cold PBS, which was placed in the tube, and centrifuged at 1500 RPM for 10 minutes at 4 °C. Pellets were washed with 500 μL of ice cold PBS to removed remaining ECM, and centrifuged at 1500 RPM for 10 minutes at 4 °C. 3 mL TrypLE (Gibco, 12605010) was added to the pellet and placed at 37 °C for 15 minutes. The pellet disrupted using “hard pipetting” (using a P1000 pipette, the organoids were pipetted quickly and vigorously). DMEM with 10% serum was added to stop trypsinisation. The organoids established as previously outlined above, and fed with RhoK inhibitor supplemented in CHFM for two days after trypsinisation, and then subsequently fed using CHFM without RhoK inhibitor.

### CLO establishment

Using early passage 2D primary cell lines, which had been established from organoids, cells were trypsinised, and a cell suspension of 5 × 10^4^ cells per well to be seeded was made, and centrifuged at 1500 RPM for 10 minutes at 4 °C. Cells were resuspended in 20 μL ECM per well, and plated and fed as outlined above. Two weeks after establishment, cells were passaged as organoids, as outlined above.

### Species confirmation PCR

DNA was extracted from organoids and primary cell lines using DNeasy Blood & Tissue Kit (Qiagen, 69504). PCRs were performed using MyTaq™ PCR Kit. The first PCR is performed using a universal primer pair complementary to conserved sequences in cytochrome B and 16S rRNA genes.

100 ng DNA, 10 pmol Primers (Table 1), 0.5 µl MyTaq HS Red Mix, 2 × 12.5 µL Water (ddH_2_O) up to 25 µl. Amplification was carried out in G-Storm, at 94 °C for 5 minutes, 59 °C for 5 minutes, followed by 35 cycles of 72 °C for 2.5 minutes, 94 °C for 30 seconds, 59 °C for 45 seconds, and followed by a final annealing step of 72 °C for 10 minutes. Samples were stored at 4 °C.

**Table 1:** Primers for PCR.

	Forward 5′−3′	Reverse 5′−3′
Universal	THGTHSAATGAATCTGAGGVGGVT	CGATGTTGGATCAGGACATC
Mouse	GCACTGAAAATGCTTAGATGGATAATTG	CCTCTCATAAACGGATGTCTAG

The amplified product from the first PCR was diluted 1:10 with ddH_2_O. 1 μL PCR product, 10 pmol Primers (Table 1), 0.5 µl MyTaq HS Red Mix, 2 × 12.5 µl Water (ddH_2_O) up to 25 µL. Amplification was carried out in G-Storm, 94 °C for 5 minutes, 60 °C for 5 minutes, followed by 30 cycles of 72 °C for 1.5 minutes, 94 °C for 30 seconds, 60 °C for 30 seconds, followed by a final annealing step of 72 °C for 10 minutes. Samples were stored at 4 °C. 5 μL of second PCR product was run on a 2% agarose (Sigma), stained with SafeView (NBS Biologicals, NBS-SV1), visualized under UV light, and imaged. A 100 bp DNA Ladder (ThermoFisher, 15628019) was applied as a size marker.

### Brightfield imaging

Brightfield imaging of PT127 CLOs and PT291 CLOs and matched organoids was performed on days 0, 3, 7, and 10 to determine the morphological changes of the cells. Pictures were taken using a Canon EOS 550D camera (Canon GmbH, Krefeld, Germany) through a Leica DM IL LED inverted microscope (Leica Microsystems, Wetzlar, Germany). Scale bars were added using ImageJ.

### Immunofluorescence

#### Cell lines

A cell suspension of 5 × 10^4^ in 500 μL was seeded in a glass bottomed 8-well plate (Ibidi, 80827), and grown overnight. Media was removed from the cells, and washed three times in a PBS 0.1% Tween 20 (Sigma, P1379) and 2% BSA (Sigma, A9418) solution. Cells were fixed in ice-cold methanol for 5 minutes at −20 °C, and washed in the PBS-T-BSA solution.

#### Organoids and CLOs

Four wells of organoids at day 7 were combined and centrifuged at 1500 RPM for 10 minutes in a 1.5 mL Eppendorf tube. All media was removed from the cell pellet, and iPGell (Funakoshi, PG20–1) was used according to the manufacturer’s protocol. Gel plugs organoids were placed in optimal cutting temperature (OCT) embedding matrix gel (Tissue-Tek, KMA-0100–00A) in a disposable histology mold (Lecia, 3803025) and placed at −80 °C overnight. Organoids were cut into 10 μm sections using a cryostat (Leica, CM 1900) which was cooled to −25 °C prior to use. OCT embedded organoids were mounted onto the freezing block using OCT, and faced off by cutting 60 μm sections until the organoids were exposed. The organoids were cut into 10 μm sections and placed onto SuperFrost Plus slides (ThermoFisher, 4591PLUS4), and stored at 4 °C until further use.

Cells were blocked for 1 hour 30 minutes in 10% Goat Serum (Gibco, 16210064), the primary antibody (Supplementary Table 5) was made in the PBS-T-BSA solution, and was added to the cells overnight at 4 °C. The cells were washed three times in the PBS-T-BSA solution, and the secondary antibody was made up in the PBS-T-BSA solution and added to the cells. The cells were incubated on a rocker for an hour at room temperature in the dark. The secondary antibody was removed, and the cells were washed three times in the PBS-T-BSA solution, DAPI (1:2500) was added to the cells for 3 minutes, and the cells were washed three times in PBS-T-BSA solution. The 24 well coverslips were mounted using ProLong Gold Antifade Mountant (Invitrogen, P36930), and allowed to dry for 24 hours. The immunofluorescence was observed using a Leica TCS SP8 STED super-resolution microscope equipped with a CCD camera and 100 × oil immersion objective. DAPI was excited with a 405 nm picoquant laser unit and emission captured between 387 and 474 nm. Alexa Fluor 488 was excited at 499 nm with emission captured between 490 and 566 nm. Images were acquired whereby combinations of excitation and emission wavelengths for specific dyes were applied sequentially.

### Quantitative reverse transcription PCR (qRT-PCR)

A High Capacity cDNA Reverse Transcription Kit (Applied Biosystems 4368814) was used to synthesis cDNA from RNA. Master mix was prepared as described in the protocol. A G-Storm Thermocycler (Model GS1, Somerton Biotechnology Centre) was used to perform cDNA synthesis at 25 °C for 10 minutes for annealing, 37 °C for 2 hours for cDNA synthesis, 85 °C for 5 minutes for enzyme inactivation, and storage at 4 °C. Following synthesis, cDNA was stored at −20 °C. TaqMan qRT-PCR was used to assess *NANOG, OCT4* and *SOX2* expression in the PDX, organoid, CLO, and cell line panels. TaqMan Gene expression assays (Thermo Scientific, *NANOG* Hs02387400; *POU5F1* Hs04260367 and *SOX2* Hs01053049), TaqMan Fast Advanced Master Mix (Applied Biosystems, 4444556), RT-PCR Grade Water (Thermo Scientific, AM9935) and cDNA template were prepared as described in the assay protocol using 20 ng of cDNA, in MicroAmp Fast Optical 96 well reaction plates (Applied Biosystems, 4346907). The plate was sealed using Adhesive PCR Plate Seals (Thermo Scientific, AB0558) and centrifuged briefly. Using an Applied Biosystems 7900 Real-Time PCR System qRT-PCR was performed using 50 °C for 2 minutes, 95 °C for 20 seconds, and 40 cycles of 95 °C for 1 second and 60 °C for 20 seconds.

### RNA-sequencing

Organoids and CLOs were grown as described for 14 days. TRI-reagent® (Sigma) was used to lyse cells, and RNA isolation was performed using Direct-zol RNA Miniprep Plus Kit (Zymo Research, R2072). RNA samples were quality controlled with an RNA Screen Tape (Agilent) and quantified with Qubit RNA HS Assay kit. Sequencing libraries were prepared with 200 ng of total RNA for all samples. The Nugen Universal Plus mRNA protocol was followed as recommended by the manufacturer. Libraries were amplified by 15 cycles of PCR. Libraries were sequenced in one NextSeq550 run with the NextSeq500/550 High Output Kit v2.5 (300 cycles), sequencing was done with Paired End 150 nt reads and 8 nt indexes. Libraries were loaded at 1.4 pM, 5% PhiX control was used.

### RNA analysis

Raw sequencing files were merged for each sample to generate a single fastq file per sample. Cutadapt was used to remove adapter sequences. Trimmed reads were aligned against the *Homo sapiens* Ch37 genome (hg19) with hisat2 using default parameters. The resulting sam file was piped into samtools for bam conversion and sorting. The resulting featureCounts object can easily be manipulated to generate a counts matrix for DESeq2. This counts generation is written to counts.tsv for further analysis. Up/down regulated genes: differential expression genes (p < 0.001 and log2 fold-change ≧ 2) are detected using DESeq2^35^ after filtering out reads count less than 10. Compare CLOs with CLs; compare PT127 PDX, CLO with CL; compare PT291 organoids, CLO with CL. The correlation of paired groups - for each sample, reads count is normalised to TPM. Median is applied if there is more than one sample in a group. Linear regression was performed to identify the relationship of paired groups: PT291 ORG and PT127 PDX; PT127 PDX and PT127 CLO; PT291 ORG and PT291 CLO, (p < 0.05). Raw and processed RNA-seq files are available at GEO (accession number GSE144737).

## Discussion

In this study, we present a method for the establishment of organoids, and isogenetically matched 2D primary cell lines from PDAC PDX tumour samples and recapitulation of the cell lines to CLOs, which we show are morphologically, molecularly and transcriptomicly similar to their PDX derived tumour organoids. This method can be utilised for all cancer types, allowing laboratories to utilise PDX samples already available for the development of organoids and primary cell lines. While PDX tumours remain stable during passaging, tumour-associated stroma is replaced with murine stromal cells^36^, with up to 90% of the cells in a PDX tumour being murine stroma^28^. When establishing primary cell lines from PDX samples, these murine fibroblasts can often quickly out-grow and outnumber the PDAC cancer cells *in vitro*, resulting in a completely unrepresentative model for PDAC^37^. In order to overcome this issue, we used mouse cell depletion kit, which results in the removal of infiltrating mouse cells from the tumour sample^33^. Utilisation of the mouse cell depletion kit to develop organoids and 2D primary cell lines from PDX tumours ensures the starting material is pure tumour tissue.

While organoids are a more representative model of PDAC *ex vivo*, they are expensive to culture, require time-consuming maintenance and can be difficult to scale up in short periods. In order to overcome these issues, we highlight the establishment of cell line organoids (CLOs). The CLOs are developed from 2D primary cell lines within two passages, grown under the same conditions as PDX organoids. These CLOs were imaged, and found to be morphologically similar to the matched organoids. To validate CLOs as representative organoid models, we molecularly profiled the expression of tumour initiating markers in our cells. Expression of ALDH1A1 and CXCR4 was low in both the PT291 and PT127 2D primary cell lines, but consistently expressed in organoid and CLOs. ALDH activity selectively defines an enhanced tumour-initiating cells^38^. ALDH1A1 is involved in retinoic acid metabolism, and has a role in proliferation and differentiation^39^. The expression of the transmembrane glycoprotein, EpCAM was retained in all models, and is known to be highly expressed in PDAC and established cell lines^40,41^.

Interestingly, PDX1 expression was low in CLOs compared to 2D primary cell lines. PDX1 is a transcription factor which is essential for pancreatic development and differentiation of all pancreatic cell lineages^42^. The over expression of PDX1 in PDAC highlighted its role as an oncogene^43^. However, PDX1 expression may be linked to the changing stages during cancer development and progression. PDX1 loss or downregulation is associated with a more aggressive phenotype, and loss of PDX1 observed in subset of cells undergoing EMT^44,45^. Furthermore, upregulation of key stem cell markers, transcription factors *NANOG*, *OCT4* and *SOX2* in CLOs compared to 2D primary cultures indicates a pluripotent stem cell state and suppression of lineage-specific genes in these models^46^.

RNA-seq analysis highlighted by PCA that the PT291 organoids and CLOs clustered together as did the PT127 PDX-tumour and CLOs compared to their respective 2D primary cell lines. Additionally, hierarchical clustering comparing the dysregulated genes between PT291 and PT127 CLOs versus the 2D primary cell lines, revealed a clear signature pattern as shown by heat map (Fig. 5B). The three top up and down regulated genes in the CLOs vs primary cell lines were *TFF3*, *PLEKHB1*, *LTB* and *FOSB*, *CAV1*, *AHNAK2*, respectively. Interestingly, *FOSB* is a proto-oncogene, and *CAV1* expression has been linked with mammary tumorigenesis and increased lung metastasis^47,48^. *AHNAK2* has also previously been found to be upregulated in PDAC^49^. The up regulated signature also contains increased expression of genes associated with cilia (*KIF12, RSPH1* and *CCDC114)*^50–52^. Cilia have been found to play an important role in modulating signalling cascades including the WNT signalling pathway, and are essential in pancreatic tissue organisation^53,54^. The upregulation of stem cell markers *ALDH1A1, ALDH1B1* and *LRIG1* may indicate restored embryonic programs and dedifferentiation pattern among our CLOs^39,55,56^. This dedifferentiation theory is supported by a previous study which has shown that when implanted orthotopically, organoids recapitulate tumour progression from precursor PanIn lesions to metastatic carcinoma^25^. Interestingly, genes down regulated in PDX tumour/organoids and CLOs include genes which have increased expression in cancers, including potential PDAC biomarkers (*CYR61, PLAU*, *FOSB, ECM1, LIF, GJB3 and CAV1)*^48,49,57–62^. There is also an increased expression of tumour suppressor genes *LRIG1* and *SEMA3F*^55,63^. This gene signature also highlights the “phenotypic switch” of the 2D the primary cell lines as the cells invade through ECM, attach and proliferate, resulting in increased motility, invasion and proliferation of cells by up-regulation of oncogenic pathways.

A limitation of our study was the use of non-metastatic surgical samples which were implanted as subcutaneous PDX tumours which did not metastasise in the mouse. In order to establish CLOs as an invasive disease model for PDAC, future work will include the establishment of preclinical models from biopsies of metastatic sites. Further limitations of our study and others studying PDAC organoids^25,46,64,65^ include the lack of a relevant microenvironment integrating the immune and stromal components, as tumour derived organoids exclusive express ductal cells, but not of other pancreatic cell lines^46^. Tsai *et al*.^66^ constructed complex organotypic models containing tumour, stromal and immune components of the tumour microenvironment. This study showed spheroids derived from established PDAC cell line, Panc-1, a mouse xenograft cell line, and the A549 lung cancer cell line are phenotypically distinct from primary organoids as they formed homogenous, non-lumen forming spheres. However, the cell line models used by Tsai *et al*.^66^ are not primary cultures, and were not compared to their isogenetically matched organoid or PDX tumour, therefore are not directly comparable and tumour to tumour bias may exist.

## Conclusions

As thirty years of cancer research have done little to improve patient outcomes, additional disease modelling strategies are required to complement existing techniques and advance PDAC research. Here, we demonstrate that CLOs established from 2D primary cell lines are a physiologically relevant *in vitro* organoid-similar model to study cancer. CLOs have the molecular and transcriptomic phenotype resembling organoids, however offer more flexibility, expandability and utility for downstream translational research and for the development of personalised treatment of PDAC patients.

## Supplementary information

Supplementary tables.
