# Supplementary material for: Modelling of pancreatic cancer biology: transcriptomic signature for 3D PDX-derived organoids and primary cell line organoid development
Source: Sci Rep. 2020 Feb 17;10:2778. doi: 10.1038/s41598-020-59368-7 (PMC7026166; doi:10.1038/s41598-020-59368-7)
Supplement: Supplementary file 1 — Supplementary tables. [file 41598_2020_59368_MOESM1_ESM.pdf]

Modelling of pancreatic cancer biology: transcriptomic signature for 3D PDX-derived organoids and primary cell line organoid development

Shannon R Nelson[1], Chenxi Chang[2,3], Sandra Roche[1], Fiona O'Neill[1], Niall Swan[4], Yonglun Luo[3,6], AnneMarie Larkin[1,5], John Crown[4], Naomi Walsh[1]*

*Correspondence to naomi.walsh@dcu.ie

[1]National Institute for Cellular Biotechnology, School of Biotechnology, Dublin City University, Dublin 9, Ireland

[2]BGI Education Centre, University of Chinese Academy of Sciences, Shenzhen 518083, China

[3]Lars Bolund Institute of Regenerative Medicine, BGI-Qingdao, BGI-Shenzhen, Qingdao, 266000, China

[4]St Vincent's University Hospital Elm Park, Dublin 4, Ireland

[5]Institute of Technology, Sligo, Ash Lane, Sligo, Ireland

[6]Department of Biomedicine, Aarhus University, 8000, Aarhus, Denmark

Supplementary Tables

Supplementary Table 1 Basic Media

**Reagent**	**Stock Concentration**	**Volume**	**Final Concentration**
DMEM, high glucose, GlutaMAX™ Supplement		100 mL	
HEPES Buffer	100X (1 M)	1 mL	1X (10 mM)
Penicillin	100X	0.25 mL	1X

Supplementary Table 2 Wash media

	**Stock Concentration**	**Volume**	**Final Concentration**
DMEM		100 mL	
HEPES Buffer (Sigma, H3375)	100X (1 M)	1 mL	1X
L-Glutamine	100X	1 mL	1X
Penicillin-Streptomycin (ThermoFisher, 15140122)	100X	1 mL	1X
FBS	100%	2.5 mL	2.5% (v/v)

Supplementary Table 3 Digestion media

**Reagent**	**Stock Concentration**	**Volume / Mass**	**Final Concentration**
Basic Media		8 mL	
Collagenase I (ThermoFisher,17018029)		40 mg	5 mg/mL
Dispase II (Sigma, D4693)		10 mg	1.25 mg/mL
FBS	100%	0.2 mL	2.50%

Supplementary Table 4 Complete Human Feeding Media (Prewarm to 37 °C)

Reagent	Stock Concentration	Volume	Final Concentration
Basic Media		20.6 mL	
Conditioned Media		25 mL	50% v/v
A83-01 (Sigma, SML0788)	1000X (0.5 mM)	50 µL	500 nM
hFGF10 (R&D, 345-FG-025/CF)	1000X (100 µg/mL)	50 µL	100 ng/mL
Gastrin I (TOCRIS, 3006)	1000X (10 µM)	50 µL	0.01 µM
N-acetylcysteine (R&D, 5619)	400X (500 mM)	125 µL	1.25 mM
Nicotinammide (Sigma, N0636)	100X (1M)	500 µL	10 mM
B-27 Supplement (Life Technologies, 17504-044)	50X	1000 µL	1X
Y-27632(Sigma,Y0503)*	1000X (10.5 mM)	50 µL	10.5 µM

**Y-27632 (Rho Kinase Inhibitor) is only necessary when organoids are first prepared, when organoids are thawed, or when organoids are dissociated to single cells.*

Supplementary table 5: Antibody conditions, secondary antibodies, suppliers and catalogue numbers for confocal microscopy .

**Antibody**	**Concentration**	**Secondary**	**Supplier**	**Catalogue number**
ALDH1A1	1:200	Rabbit	Abcam	ab52492
CXCR4	1:100	Mouse	SCBT	sc-53534
EpCAM	1:50	Mouse	SCBT	sc-25308
PDX1	1:50	Rabbit	Invitrogen	PA514824
Alexa Fluor 488 Mouse	1:1500	-	ThermoFisher	A-11001
Alexa Fluor 488 Rabbit	1:2000	-	ThermoFisher	A-11008

Supplementary table 6: Gene list – genes upregulated in both PT127 PDX and CLO when compared with PT127 cell line (fold change ≥2, p-adj= 0.001)

Gene Name	log2FoldChange	padj
KIF12	5.422	1.62E-62
CA12	5.302	1.44E-19
LRIG1	5.286	5.37E-57
LTB	5.263	8.48E-05
FAM3B	5.004	6.01E-51
TNFRSF11B	4.760	5.34E-09
SLC5A1	4.749	1.95E-50
LCN2	4.551	4.24E-12
PLEKHB1	4.505	4.08E-13
DEGS2	4.455	1.86E-07
FAM222A	4.176	1.96E-08
NECTIN3	3.920	4.77E-14
SELENBP1	3.873	1.29E-12
GJB1	3.831	1.06E-12
FILIP1L	3.795	1.92E-14
MYH7B	3.776	5.46E-07
PLB1	3.748	2.16E-24
NR0B2	3.738	4.10E-09
PLEKHS1	3.669	3.63E-28
PLA2G4F	3.638	1.05E-24
CLDN2	3.594	4.09E-79
SLC9A3	3.539	9.80E-12
C9orf152	3.527	7.66E-12
SMIM24	3.516	3.49E-09
KLK1	3.488	8.79E-23
CCDC40	3.452	1.39E-11
RSPH1	3.418	1.59E-45
PSTPIP2	3.394	4.18E-08
ITPR2	3.386	5.38E-07
BRSK2	3.365	0.00015
PTGER4	3.344	1.79E-19
DAPK2	3.338	6.65E-10
LINC01124	3.277	1.35E-07
ZSWIM5	3.245	5.34E-20
AMACR	3.235	1.17E-18
IGSF9	3.175	2.46E-18
SEMA6A	3.160	1.90E-06
MEX3A	3.125	2.64E-08
DEPTOR	3.115	2.43E-52
EPHX2	3.031	2.45E-18
LARGE2	2.996	5.92E-06
TMEM229B	2.979	9.77E-13
100288152	2.975	3.29E-08
MAP2K6	2.963	4.38E-10
ATP10B	2.953	7.15E-42
GPX2	2.941	1.01E-31
SLC1A4	2.927	1.58E-16
CRACR2A	2.925	1.25E-05
RNASE1	2.913	1.44E-08
SELENOM	2.889	1.28E-21
RXFP4	2.882	2.53E-09
RGMB	2.871	3.57E-18
SEZ6L2	2.867	1.46E-07
SLC4A8	2.855	6.12E-06
MLLT3	2.845	1.31E-19
BDH2	2.839	3.17E-10
EPHB3	2.809	6.31E-11
CRISPLD2	2.797	0.00023
PDZK1	2.786	6.55E-13
TPPP3	2.755	3.95E-06
SP5	2.753	0.00019
CCDC170	2.751	1.43E-12
MDK	2.746	1.34E-10
PAQR6	2.738	0.00074
MAP1LC3A	2.730	2.32E-14
RAB30	2.718	1.91E-05
SOWAHA	2.699	2.31E-14
NEDD9	2.694	2.88E-05
ACSS1	2.682	5.28E-20
MYCL	2.673	5.40E-09
BCL2L14	2.646	0.0002
HNF1A-AS1	2.643	5.24E-06
GPC2	2.640	1.22E-06
SIAE	2.640	4.95E-06
100124692	2.634	5.42E-34

KREMEN1	2.604	4.31E-06
TSPOAP1	2.579	1.86E-08
SELENOW	2.579	1.13E-18
INPP5J	2.543	7.16E-07
IFI6	2.534	2.67E-05
MCF2L	2.533	2.81E-10
RAB26	2.519	4.82E-05
MGAM2	2.517	3.09E-07
INKA2	2.505	2.42E-14
ORMDL3	2.505	2.02E-09
MAPK8IP1	2.498	2.49E-06
TNFAIP2	2.496	1.92E-08
GRAMD2A	2.490	5.35E-16
TGFB3	2.452	0.00093
PDE4D	2.445	1.06E-08
TOX3	2.440	0.00029
ALDH1B1	2.438	8.55E-53
HS6ST1	2.417	6.87E-08
SLC29A4	2.400	0.00014
AIFM3	2.394	6.63E-10
ARHGEF40	2.394	3.02E-05
SCAMP5	2.376	1.26E-12
FAAH	2.375	5.63E-10
KCTD17	2.360	1.83E-06
FOXA3	2.360	1.43E-12
ETS2	2.352	2.60E-32
GSDMB	2.327	0.00041
SGSH	2.324	4.02E-08
PAPSS2	2.323	0.00042
PYCARD	2.312	1.55E-06
ASRGL1	2.276	7.24E-08
FKBP7	2.267	0.00013
PLPP3	2.266	0.00066
ALDH1A1	2.259	2.75E-46
GATM	2.258	4.67E-09

ABAT	2.244	2.45E-10
SOX9-AS1	2.227	8.80E-05
FOXD4	2.209	1.25E-05
ESPN	2.190	2.55E-09
MYLIP	2.189	1.25E-25
CCSER1	2.187	0.00064
VIPR1	2.178	7.25E-14
RN7SK	2.167	1.06E-08
SERPINA4	2.162	1.75E-07
IFI27	2.151	0.00094
CEMIP2	2.148	1.16E-07
TESC	2.138	2.12E-06
RBBP8NL	2.134	4.95E-17
MMP11	2.129	3.08E-08
CES3	2.122	1.97E-11
AKR1C3	2.118	2.76E-16
STXBP6	2.107	3.33E-11
MLEC	2.102	1.18E-11
QSOX1	2.095	1.20E-14
NFIA	2.086	2.62E-12
CHST6	2.070	2.07E-11
ARRB2	2.067	3.82E-16
STK31	2.063	9.70E-06
CTSS	2.063	1.18E-08
ZNF233	2.050	5.37E-05
LRRC73	2.048	1.69E-05
MYB	2.047	1.51E-14
RAB11FIP3	2.044	3.49E-05
100129046	2.029	3.29E-14
TEAD2	2.025	2.91E-17
PLCB4	2.025	0.000444
CORO1A	2.019	1.13E-31
ACCS	2.006	7.84E-05
SYK	2.002	1.96E-08

Supplementary table 7: Gene list – genes downregulated in both PT127 PDX and CLO when compared with PT127 cell line (fold change ≥2, p-adj= 0.001

Gene List	log2FoldChange	padj
FOSB	-7.241	1.82E-55
CAV1	-7.054	1.16E-101
AXL	-6.625	6.32E-262
RAB3B	-6.322	1.39E-60
FOS	-5.132	1.13E-31
PADI1	-4.767	1.24E-51
DUSP1	-4.670	2.09E-42
EHD2	-4.651	2.54E-09
EGR1	-4.637	3.22E-50
ATF3	-4.383	4.50E-09
TGM2	-4.348	3.35E-176
CYR61	-4.301	1.62E-62
OXTR	-4.236	1.98E-10
PHACTR3	-4.210	1.13E-31
NR4A1	-4.174	6.61E-11
FOSL1	-3.986	2.47E-35
KRT86	-3.876	1.44E-19
SUSD2	-3.862	4.44E-26
AKAP12	-3.796	1.86E-64
ADGRF1	-3.731	9.83E-27
LCK	-3.681	2.45E-21
CAVIN1	-3.660	1.43E-78
PLK2	-3.623	2.30E-40
LGALS1	-3.598	4.75E-45
284454	-3.584	1.52E-09
ARHGDIB	-3.580	2.63E-34
EDN1	-3.579	1.43E-64
UNC13D	-3.472	1.51E-27
MMP7	-3.463	1.17E-08
ELFN2	-3.455	1.30E-06
ANXA1	-3.415	4.82E-31
CACNG4	-3.391	2.59E-18
PLAU	-3.355	5.27E-60
KLF2	-3.347	1.90E-17
CAV2	-3.346	4.90E-24
GPAT3	-3.297	3.55E-28
HBEGF	-3.295	1.56E-07
MYBL1	-3.270	5.46E-06
CTGF	-3.252	4.80E-14
ERRFI1	-3.243	6.42E-14
MICB	-3.203	1.24E-11
SMAD7	-3.144	2.10E-10
TSC22D3	-3.136	1.27E-12
THSD4	-3.083	2.45E-21
KRT80	-3.059	9.81E-55
SLC7A7	-3.044	1.05E-16
PMAIP1	-3.024	3.98E-15
652995	-3.020	5.44E-17
ID2	-3.014	2.31E-11
RGCC	-2.952	1.82E-29
L1CAM	-2.873	2.27E-07
PLAUR	-2.868	5.74E-06
GADD45B	-2.860	8.18E-09
SLC16A4	-2.814	8.15E-26
ARHGAP29	-2.813	2.58E-08
LAT2	-2.792	3.03E-32
PEAR1	-2.791	2.23E-05
FGFBP1	-2.787	0.00016
ANO1	-2.774	1.11E-36
ZFP36	-2.761	1.81E-05
WNT7B	-2.753	2.44E-11
NCEH1	-2.711	4.27E-23
NTN4	-2.633	6.66E-05
MIR4435-2HG	-2.628	7.08E-32
STEAP4	-2.623	3.44E-21
SNORC	-2.621	9.24E-14
CAVIN3	-2.618	5.03E-16
ANXA2R	-2.603	4.38E-10
CYTOR	-2.583	5.81E-09
RNF39	-2.571	1.95E-19
KLF6	-2.552	2.57E-05
ANLN	-2.544	3.89E-17
KLK7	-2.532	0.0007
ID3	-2.517	2.16E-05
ERFE	-2.493	6.50E-08

100287314	-2.489	7.83E-16
SGK1	-2.486	7.57E-21
TMPRSS13	-2.476	0.00031
CLCF1	-2.457	2.64E-08
ATP2B4	-2.452	1.11E-13
DUSP10	-2.448	1.77E-39
IL6R	-2.393	3.62E-24
OLFML2A	-2.376	4.05E-07
FAM122B	-2.376	2.04E-12
MSMO1	-2.363	3.79E-09
SMURF2	-2.356	6.07E-16
TNNT1	-2.347	2.70E-08
DCBLD2	-2.337	1.31E-05
221584	-2.336	9.97E-10
CHAC1	-2.331	4.38E-07
DUSP6	-2.327	1.22E-19
LIF	-2.319	4.58E-05
MOSPD1	-2.318	2.88E-09
ARNTL2	-2.299	4.73E-07
ZNF165	-2.291	1.88E-33
FAM122C	-2.291	5.25E-15
643977	-2.289	1.15E-13
TIMP2	-2.286	9.19E-49
ARSK	-2.263	1.54E-05
F3	-2.255	1.34E-07
SEMA7A	-2.223	2.26E-06
CDKN2B	-2.212	1.41E-05
100506906	-2.206	0.00088

EVA1C	-2.205	7.97E-24
DUSP5	-2.190	0.00055
LAT	-2.182	1.07E-14
BCAR3	-2.176	9.14E-32
ITGB2	-2.171	1.16E-07
CXCL2	-2.162	1.06E-06
GRHL3	-2.145	2.98E-23
TTYH1	-2.144	1.43E-05
CITED2	-2.143	1.99E-29
HMGA1	-2.140	1.89E-15
PTAFR	-2.139	4.88E-19
B3GNTL1	-2.132	6.95E-11
AMIGO2	-2.130	1.33E-17
KRT7	-2.110	0.00060
RBMS2	-2.096	2.03E-10
FAM3C	-2.078	7.55E-22
SLC20A1	-2.068	1.79E-07
HES1	-2.066	2.02E-15
DENND3	-2.063	1.00E-06
KIAA0040	-2.047	0.00049
HDAC9	-2.046	3.15E-13
VSIR	-2.044	1.01E-09
THBS1	-2.041	8.25E-18
S100A2	-2.015	4.64E-06
KIF20B	-2.011	0.00038
HMGCS1	-2.005	2.24E-07
ETS1	-2.000	2.01E-05

Supplementary table 8: Gene list – genes upregulated in both PT291 PDX and CLO when compared with PT291 cell line (fold change ≥2, p-adj= 0.001)

Gene Name	log2FoldChange	padj
SERPINA5	4.212	2.17E-14
CCDC114	4.170	4.30E-22
PTK7	4.061	2.95E-27
FCGBP	3.931	1.87E-29
CCDC170	3.607	4.53E-13
ANPEP	3.575	1.43E-09
TFF3	3.512	3.51E-11
NECTIN3	3.469	4.17E-09
NDRG1	3.461	1.94E-11
SERPINA4	3.414	2.62E-13
PPIL6	3.215	9.37E-07
FOXJ1	3.037	2.97E-10
TGFB3	3.015	8.47E-19
SLC29A4	2.986	3.75E-07
PLEKHB1	2.890	2.69E-14
CFAP73	2.840	4.88E-07
DEPTOR	2.811	2.36E-09
EGLN3	2.794	1.90E-22
GCNT1	2.778	1.82E-09
PPM1L	2.771	3.59E-12
LRIG1	2.709	8.57E-11
FKBP9P1	2.685	5.22E-05
PRSS33	2.646	0.00026
TRPM5	2.596	0.00055
100506211	2.581	3.46E-09
CCDC40	2.580	4.78E-08
WDR78	2.576	2.32E-06
GNAZ	2.512	8.39E-10
FAM222A	2.509	1.38E-07
LINC00261	2.500	4.31E-05
ASRGL1	2.444	2.60E-06
RCBTB2	2.434	2.55E-11
SEMA3F	2.362	2.31E-23
STXBP6	2.343	1.99E-11
TMEM97	2.316	0.00070
LRRC73	2.315	2.94E-11
ITPR2	2.306	1.32E-05
NAALADL2	2.272	5.79E-14
RASL11A	2.272	7.44E-05
APBA1	2.271	4.59E-08
FHAD1	2.271	6.05E-10
FAM227A	2.262	5.65E-10
CLU	2.239	1.20E-05
RNASE1	2.230	4.96E-05
DEGS2	2.214	1.16E-08
RSPH1	2.202	2.63E-08
HNF1A-AS1	2.194	9.64E-07
PTPRN2	2.192	3.33E-13
SYNM	2.179	1.02E-05
C4A	2.176	7.21E-08
PPP1R1B	2.151	1.64E-20
USP2	2.146	6.50E-06
TMEM231	2.144	1.60E-05
C4B	2.131	8.57E-08
ST6GALNAC1	2.128	4.31E-05
MESP1	2.083	6.24E-09
SNED1	2.082	2.69E-13
ALDOC	2.066	0.00069
PROM1	2.050	1.70E-12
HIST1H4K	2.043	1.65E-06
SLC4A8	2.042	2.91E-07
FAM110C	2.018	1.47E-06

Supplementary table 9: Gene list – genes downregulated in both PT291 PDX and CLO when compared with PT291 cell line (fold change ≥2, p-adj= 0.001)

Gene Name	log2FoldChange	padj
AHNAK2	-5.316	1.28E-110
AXL	-5.301	2.00E-44
COL17A1	-4.747	0.0002
CDHR2	-4.682	4.55E-11
ECM1	-4.628	9.92E-49
FOSB	-4.491	2.55E-11
ID3	-4.263	8.26E-17
SEMA7A	-3.985	5.67E-39
IL23A	-3.926	1.92E-29
AKAP12	-3.887	3.40E-11
TM4SF4	-3.865	0.00055
FLNC	-3.848	1.75E-08
CDH17	-3.757	0.00044
LAT2	-3.757	2.11E-46
CAVIN1	-3.704	8.30E-24
UNC13D	-3.629	8.24E-24
SNCG	-3.594	4.14E-08
SULT2B1	-3.551	1.25E-11
LGALS1	-3.437	1.46E-26
BTBD11	-3.424	6.91E-09
RAB3B	-3.422	1.57E-10
FOSL1	-3.414	2.08E-10
PADI1	-3.414	1.48E-10
SDCBP2	-3.338	1.90E-08
CAV1	-3.321	3.16E-08
ID1	-3.267	3.22E-07
AREG	-3.230	2.10E-48
DUSP1	-3.224	2.53E-16
727738	-3.172	2.10E-48
F3	-3.047	9.10E-15
CPNE7	-2.962	2.07E-06
ABLIM3	-2.933	2.95E-27
CYP4F3	-2.925	0.00035
PLAU	-2.918	1.10E-17
SMAD7	-2.908	3.01E-18
AMIGO2	-2.875	3.99E-21
GPAT3	-2.828	6.09E-09
PHACTR3	-2.787	5.07E-06
EMP1	-2.774	6.03E-25
GJB3	-2.763	2.63E-34
PLAUR	-2.757	3.59E-17
MDFI	-2.757	2.68E-12
GEM	-2.736	2.77E-18
IL18	-2.683	1.84E-10
652995	-2.681	2.51E-05
DCBLD2	-2.678	8.37E-11
ELFN2	-2.639	1.03E-07
SMIM5	-2.634	0.00094
SDR16C5	-2.617	9.01E-06
OXTR	-2.611	9.83E-06
PCED1B	-2.605	7.03E-05
CYP3A5	-2.588	1.02E-05
RASA3	-2.555	1.78E-06
GPSM1	-2.514	0.00032
UNC5B	-2.485	0.0006
PLEKHN1	-2.468	2.97E-10
P2RY2	-2.443	3.57E-07
ETS1	-2.436	8.63E-08
CRYBG2	-2.398	4.15E-06
ARL4C	-2.394	4.33E-09
TNFAIP3	-2.394	8.60E-05
KLK11	-2.391	3.57E-07
CXCL8	-2.391	9.14E-10
DUSP10	-2.375	1.28E-07
IL32	-2.373	1.11E-06
8511	-2.352	8.08E-05
ADAMTSL5	-2.340	1.17E-08
PTPRR	-2.336	5.46E-09
SYNGR3	-2.310	0.00077
PSORS1C1	-2.296	1.16E-06
PHLDB2	-2.277	1.48E-09
MMP7	-2.247	1.24E-06
CD55	-2.236	1.34E-13
MMP23B	-2.226	7.11E-05
ITGA2	-2.221	7.93E-26
STOM	-2.217	2.66E-07

KIAA0040	-2.189	1.50E-06
MTMR11	-2.186	1.81E-05
PERM1	-2.178	4.58E-06
PROCR	-2.177	9.52E-11
HEPH	-2.163	4.92E-05
IRS2	-2.145	1.60E-15
KLK7	-2.129	0.00032
SH3TC2	-2.120	1.15E-19
PLCXD2	-2.119	0.00030
PTPRB	-2.114	1.28E-24
LIF	-2.103	1.34E-34
PRDM1	-2.102	2.46E-07
PIM1	-2.094	2.07E-09
RND1	-2.089	0.00030
TNFRSF11B	-2.084	8.31E-11

SERPINB5	-2.083	8.15E-05
SLC22A3	-2.081	7.58E-15
S100A2	-2.070	3.58E-06
DUSP5	-2.058	4.04E-08
ARL14.00	-2.058	0.0002
FSCN1	-2.054	0.00066
284454	-2.053	2.58E-10
KLK10	-2.043	4.77E-07
CLCF1	-2.041	1.10E-06
LMTK3	-2.035	0.00051
SMIM6	-2.019	2.29E-05
CAV2	-2.012	1.42E-14
TNFRSF6B	-2.006	9.01E-05
EPHB6	-2.004	2.91E-07

Supplementary Figures

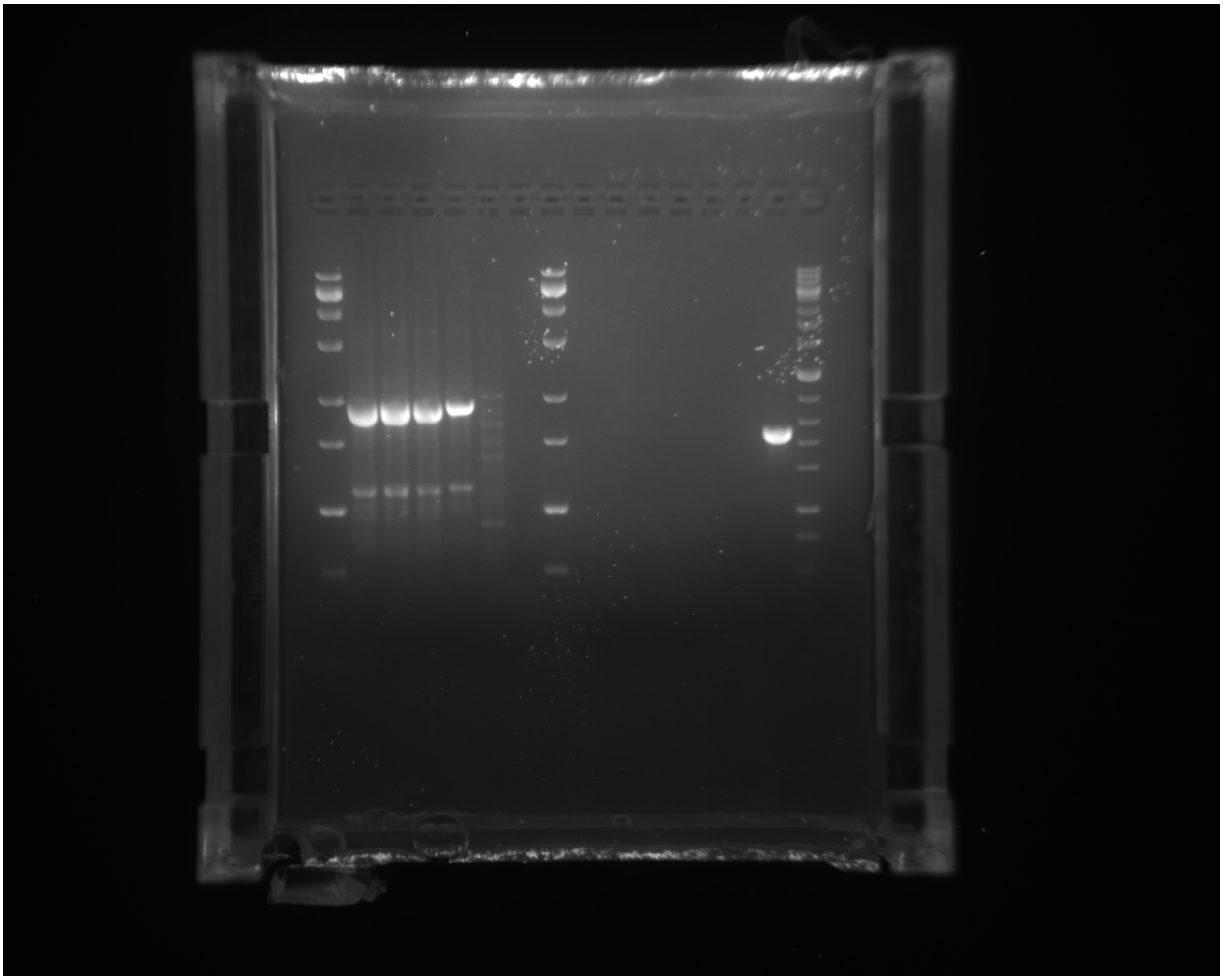

Supplementary figure 1: Entire blot from figure 3 Electrophoresis gels of samples (1) PT291 primary cell line (2) PT127 primary cell line (3) PT291 organoids (4) BxPC3 (negative control) (5) Murine cell line C2C12 (positive control) for (A) Products of first PCR and (B) Nested PCR using mouse-specific primers
